# The Major Autoantibody Epitope on Factor H in Atypical Hemolytic Uremic Syndrome Is Structurally Different from Its Homologous Site in Factor H-related Protein 1, Supporting a Novel Model for Induction of Autoimmunity in This Disease*

**DOI:** 10.1074/jbc.M114.630871

**Published:** 2015-02-06

**Authors:** Arnab Bhattacharjee, Stefanie Reuter, Eszter Trojnár, Robert Kolodziejczyk, Harald Seeberger, Satu Hyvärinen, Barbara Uzonyi, Ágnes Szilágyi, Zoltán Prohászka, Adrian Goldman, Mihály Józsi, T. Sakari Jokiranta

**Affiliations:** From the ‡ Department of Bacteriology and Immunology, Medicum, and Immunobiology Research Program Unit, University of Helsinki and Helsinki University Hospital, 00014 University of Helsinki, Finland,; the § Institute of Biotechnology and; ** Division of Biochemistry and Biotechnology, Department of Biosciences, University of Helsinki, 00014 University of Helsinki, Finland,; the ¶ Junior Research Group for Cellular Immunobiology, Leibniz Institute for Natural Product, Research and Infection Biology-Hans Knöll Institute, 07745 Jena, Germany,; the ‖ Research Laboratory, 3rd Department of Internal Medicine, Semmelweis University, 1125 Budapest, Hungary, and; the ‡‡ MTA-ELTE Immunology Research Group and; §§ MTA-ELTE “Lendület” Complement Research Group, Department of Immunology, Eötvös Loránd University, 1117 Budapest, Hungary

**Keywords:** Autoimmune Disease, Autoimmunity, Immunoglobulin G (IgG), Immunology, X-ray Crystallography, Thrombotic Microangiopathy, Atypical Hemolytic Uremic Syndrome, Complement Regulation, Structure-Function Study

## Abstract

Atypical hemolytic uremic syndrome (aHUS) is characterized by complement attack against host cells due to mutations in complement proteins or autoantibodies against complement factor H (CFH). It is unknown why nearly all patients with autoimmune aHUS lack CFHR1 (CFH-related protein-1). These patients have autoantibodies against CFH domains 19 and 20 (CFH_19–20_), which are nearly identical to CFHR1 domains 4 and 5 (CFHR1_4–5_). Here, binding site mapping of autoantibodies from 17 patients using mutant CFH_19–20_ constructs revealed an autoantibody epitope cluster within a loop on domain 20, next to the two buried residues that are different in CFH_19–20_ and CFHR1_4–5_. The crystal structure of CFHR1_4–5_ revealed a difference in conformation of the autoantigenic loop in the C-terminal domains of CFH and CFHR1, explaining the variation in binding of autoantibodies from some aHUS patients to CFH_19–20_ and CFHR1_4–5_. The autoantigenic loop on CFH seems to be generally flexible, as its conformation in previously published structures of CFH_19–20_ bound to the microbial protein OspE and a sialic acid glycan is somewhat altered. Cumulatively, our data suggest that association of CFHR1 deficiency with autoimmune aHUS could be due to the structural difference between CFHR1 and the autoantigenic CFH epitope, suggesting a novel explanation for CFHR1 deficiency in the pathogenesis of autoimmune aHUS.

## Teaser

**Background:** It is unknown why patients with autoantibodies against complement factor H (CFH) lack homologous CFHR1 protein.

**Results:** The autoantibody epitope on CFH was identified, and the structure of the corresponding part of CFHR1 was solved.

**Conclusion:** The autoantigenic epitope of CFH and its homologous site in CFHR1 are structurally different.

**Significance:** A plausible explanation for formation of autoantibodies due to CFHR1 deficiency in autoimmune atypical hemolytic uremic syndrome was obtained.

## Introduction

Atypical hemolytic uremic syndrome (aHUS)^3^ is a rare and often fatal systemic disease characterized by hemolytic anemia, thrombocytopenia, microvascular thrombosis, and kidney failure (1). It is associated with dysregulation of complement activation via mutations, polymorphisms, or rearrangements in genes coding for various complement proteins (2). The mutations are found mainly in the gene coding for complement factor H (CFH) (3, 4), which mediates elimination of the central complement activation component C3b. We and others (5–8) have shown how mutations in CFH domains 19 and 20 (CFH_19–20_) cause impaired regulation of C3b on host cells, leading to complement attack against red blood cells, platelets, and endothelial cells as seen clinically in aHUS. However, some aHUS cases are caused by autoantibodies against CFH (CFH-AAs). These antibodies have been identified in 5–11% of aHUS patients in different cohorts (9–13), but even 56% of 246 HUS patients have been reported with CFH-AAs in India (14). In nearly all of the cases, the patients have autoantibodies against the C terminus of CFH, although usually in addition to such antibodies, some patients have autoantibodies against other parts of CFH as well (10, 15). Patients with the autoimmune form of aHUS nearly always lack certain CFH-related proteins, primarily CFHR1 (CFH-related protein-1) (10, 16).

**FIGURE 1.**
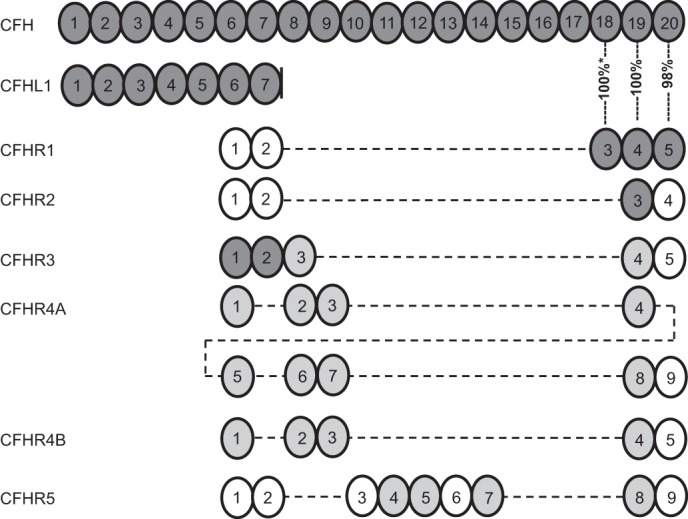
**Schematic illustration indicating the amino acid sequence identity of CFH to other members of the CFH family.** Each CFHR or CFHL domain is shown below the domain of CFH to which it has the highest amino acid sequence identity. For sequence identities of 32–49%, the domains are shown in *white*; for 57–84% identity, in *light gray*; and for 85–100% identity, in *dark gray*. The identity between domains 3–5 of CFHR1 and domains 18–20 of CFH is indicated as a percentage. The *asterisk* indicates that the sequence identity of domain 3 in the basic isoform of CFHR1 to domain 18 in CFH is 100%, whereas that of the acidic isoform is 95% (12). CFHL-1 (CFH-like molecule-1) is an alternatively spliced transcript from the *CFH* gene with four unique residues following domain 7.

CFH and CFHR proteins are encoded by adjacent genes and form a protein family (Fig. 1) (17). CFHR proteins are composed of four to nine complement control protein domains, also called short consensus repeats, and the two most C-terminal domains have relatively high sequence homologies to the C-terminal domains of CFH (Fig. 1) (17). There are only two residues that are different in the last two C-terminal domains of CFH and CFHR1 (domains 19–20 and 4–5, respectively). The functional importance of these minor differences is obvious because the hybrid *CFH*/*CFHR1* genes producing fusion proteins CFH_1–18_/CFHR1_4–5_ and CFHR1_1–3_/CFH_19–20_ have been found in aHUS patients in the absence of other mutants or CFH-AAs (4, 18–20). Domains 19 and 20 of CFH are responsible for directing its complement regulatory activity to cell and extracellular matrix surfaces by binding simultaneously to both C3b and negatively charged glycosaminoglycans or sialic acid glycans on the surfaces (6, 21, 22). The autoantibodies of nearly all patients with autoimmune aHUS recognize the C terminus of CFH, and inhibit the physiological CFH-mediated protection of host cells from complement attack (10, 11, 13, 15, 23).

More than 90% of patients with CFH-AAs lack CFHR1 and CFHR3, resulting from a homozygous deletion of the genomic region containing both of them (10, 12, 13, 16). Some patients have other rarer genetic alterations, including a homozygous *CFHR1*/*CFHR4A* deletion (12), a combination of heterozygous *CFHR1*/*CFHR3* and *CFHR1*/*CFHR4A* deletions (12, 13), or a combined heterozygous *CFHR1*/*CFHR3* deletion in the presence of a missense mutation in *CFHR1* (12). The common feature in these genetic alterations is a deficiency of CFHR1 (24, 25). However, CFH-AAs have also been described, although rarely, in patients with two normal copies of *CFHR1* and *CFHR3* but mutations in *CFH*, *CFI*, *CD46*, or *C3* genes (12, 13). CFH-AAs often cross-react with CFHR1 (13, 15, 26), but the exact location of the autoantibody site on CFHR1 has not been determined. On the basis of inhibition of autoantibody binding to CFHR1 by mAb C18 (26) and the sequence homology to the C terminus of CFH, it is likely, however, that the autoantibody-binding site is within the last two domains of CFHR1, *i.e.* far away from its N-terminal dimerization site (27).

To date, the reason for the association between CFH-AAs and CFHR1 deficiency has been unknown. In this study, we aimed to solve why a deficiency of one molecule (CFHR1) predisposes to autoimmunity against another, highly homologous molecule (CFH) in aHUS. We mapped the binding sites of CFH-AAs within CFH_19–20_ and compared the CFH-AA-binding sites with the previously reported ligand-binding sites on CFH_19–20_. Because the autoantibody epitopes formed a cluster next to the residues that are different in the two C-terminal domains of CFH and CFHR1, we decided to solve and analyze the structure of CFHR1_4–5_ and to study the potential differences in antigenicity of those two molecules. We found structural differences in the autoantibody-binding site of CFH domain 20 and the corresponding homologous site of CFHR1 domain 5. Based on these data, a novel model is proposed, suggesting how immunization against CFH domain 20 could be linked to CFHR1 deficiency.

## EXPERIMENTAL PROCEDURES

### 

#### 

##### Proteins

Cloning, expression, and purification of WT CFH_19–20_ and mutant proteins with the 14 single-point mutations have been described previously (5, 7, 28). Proper folding of the constructs was verified for three mutants (Q1139A, R1203A, D1119G/Q1139A) by solving the structures by x-ray crystallography (6, 28, 29) and for five mutants (R1182A, W1183L, K1188A, E1198A, and R1206A) by circular dichroism (30).

CFHR1_4–5_ was generated by site-directed mutagenesis of CFH_19–20_-encoding DNA in the pPICZαB vector (Invitrogen). The primer used to introduce the S1191L and V1197A mutations was CAG AAG CTT TAT TTG AGA ACA TCA GGT GAA GAA GCT TTT GTG. The mutations were confirmed by sequencing before expression of CFHR1_4–5_ in *Pichia pastoris* (strain X-33) using 1% methanol induction as described previously (7).

Recombinant CFHR1, CFH_1–7_, CFH_8–14_, CFH_15–20_, and CFHR4B were generated as described previously (31, 32). CFH was purchased from Merck. mAb C18 (33) was purchased from Enzo Life Sciences (Lörrach, Germany).

##### Patients and Blood Samples

The studies were approved by the Research Ethics Committee of the Medical Faculty of Friedrich Schiller University Jena and were performed in accordance with the Declaration of Helsinki. Patients with aHUS were screened for CFH-AAs using ELISA as described (9, 11). All patients lacked the *CFHR1* and *CFHR3* genes and proteins except for patient 2, who had two copies of both genes, and patient 10, who carried a homozygous *CFHR1* deletion and was heterozygous for *CFHR3*. Three of the patients (patients 3, 7, and 8) have been described previously (23). The characteristics of the sera containing CFH-AAs from the 17 patients are summarized in Table 1.

##### Microtiter Plate Assays

WT or mutant CFH_19–20_ proteins (5 μg/ml) were coated onto Nunc MaxiSorp plates (Thermo Scientific). Binding of patient sera (1:50–1:200 depending on the antibody titer) was analyzed as described (11). Binding of mAb C18 (5 μg/ml) was detected using peroxidase-conjugated swine anti-mouse IgG.

Binding of patient CFH-AAs (sera diluted 1:100) to CFH, its recombinant fragments, CFHR1, and CFHR1_4–5_ was compared as described above using 250 nm immobilized recombinant proteins, plasma-purified CFH (65 nm), or recombinant CFHR4B and albumin as negative controls. Relative binding was calculated from representative data sets performed in triplicates. Equal binding of anti-CFH polyclonal antiserum and negligible binding of anti-IgG to the CFH_19–20_ mutants were confirmed by ELISA.

The assay comparing the binding avidity of CFH_19–20_ and CFHR1_4–5_ with purified IgG from patient 11 was done after coating CFH_19–20_ (10 μg/ml) onto Nunc MaxiSorp plates. After blocking (0.05% Tween 20 in PBS) for 120 min and washing (0.02% Tween 20 in PBS), 40 μl of patient IgG (3.8 μg/ml) and CFH_19–20_, CFHR1_4–5_, or CFH_5–7_ were added in different dilutions and incubated for 120 min at 37 °C, followed by measuring IgG binding as described (11). The experiment was performed three times in triplicates, and the background subtracted data were normalized using the values obtained without an inhibitor (100% binding).

##### Crystallization and Solving the CFHR1_4–5_ Structure

CFHR1_4–5_ was crystallized at 293 K from hanging drops in the presence of 2 m ammonium sulfate and 0.1 m sodium acetate at pH 4.6. The cube-shaped crystals appeared within 5 days and were cryoprotected with 25% glycerol (supplemented with the mother liquor). The diffraction data (to 2.9 Å) were collected at European Synchrotron (ESRF) beamline ID14-4 (34) at 100 K on an ADSC Q315r charge-coupled device detector at 0.979520 Å. The data were indexed and scaled using XDS (35). The structure of CFH_19–20_ mutant R1203A (Protein Data Bank code 3KZJ (28)) was used as a search model in Phaser (36), and two molecules of CFHR1_4–5_ were identified in the asymmetric unit. After successive rounds of model building with Coot (37) and refinement using phenix.refine software (38), we could refine the structure to *R*_work_/*R*_free_ = 0.20/0.26 (see Table 2). The last refinement cycles were done using TLS parameters (10 TLS groups). In the Ramachandran plot, 95% of the amino acid structures were within the most favored region.

The superpositions of different structures and structural illustrations were prepared using PyMOL software (Schrödinger, Portland, OR). The surface charge distribution of both the CFHR1_4–5_ and CFH_19–20_ molecules was calculated using APBS (39), and the potentials on the solvent-accessible surfaces were displayed in PyMOL.

##### Statistical Analyses

Values are expressed as means ± S.E. using GraphPad Prism software (version 6). All curves and bar diagrams were made using the same software.

## RESULTS

### 

#### 

##### Mapping the Autoantibody-binding Residues on CFH_19–20_

**FIGURE 2.**
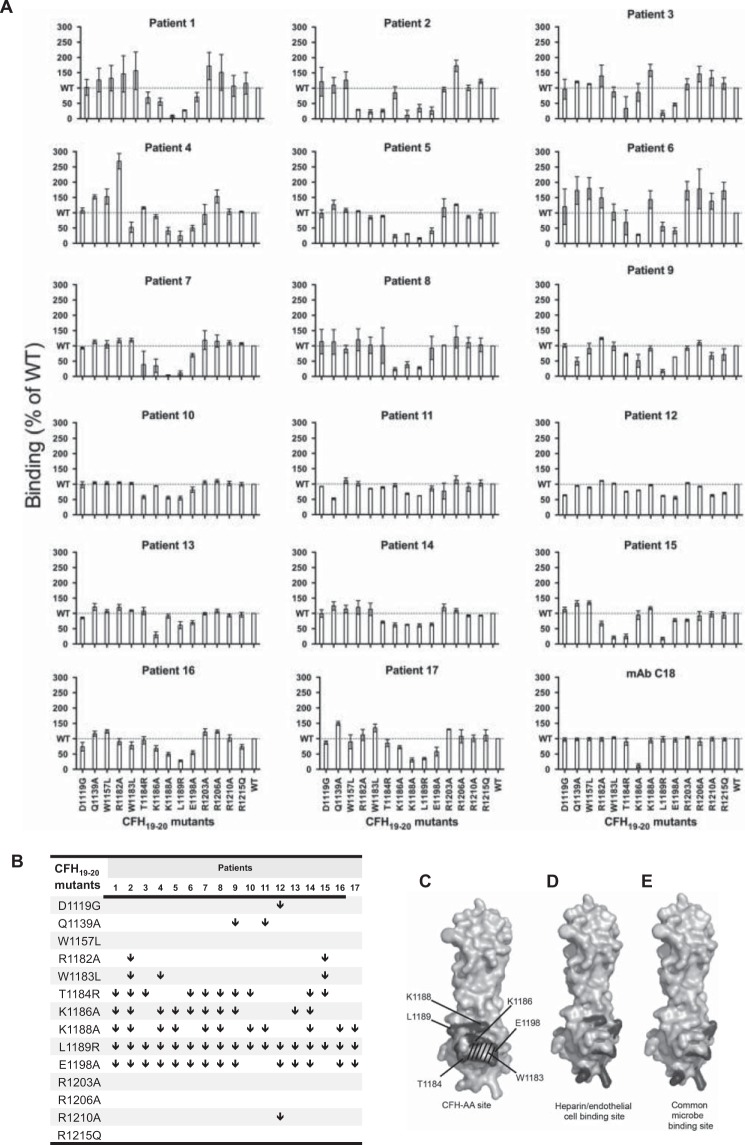
**Mapping of the CFH-AA-binding region on CFH_19–20_.** *A*, binding of IgG from 17 aHUS patients and mAb C18 to 14 CFH_19–20_ constructs with various single-point mutations in relation to binding to WT CFH_19–20_. *Error bars* indicate S.E., and the level of WT binding is indicated by *dotted lines. B*, comparison of the autoantibody-binding epitopes by identification of the mutations that impaired binding of patient IgG by at least 30% (indicated by ↓). *C*, locations of the residues involved in binding of autoantibodies to CFH_19–20_ as indicated in *dark gray* and annotated on a previously published structure of CFH_19–20_ (Protein Data Bank code 2G7I) (7). The location of Trp-1183 is indicated by *stripes. D* and *E*, for comparison, the locations of residues involved in binding of CFH_19–20_ to heparin (5, 40) (*D*) and in the common microbe-binding site on CFH_19–20_ (30) (*E*) are indicated in *dark gray*.

The binding of IgG from sera of 17 aHUS patients with CFH-AAs to 14 different CFH_19–20_ mutants was tested (Fig. 2*A*). The binding data indicated a congruent tendency as follows. The binding of IgG from all patients to the L1189R mutant was diminished by at least 30% compared with WT CFH_19–20_. IgG from 14 patients showed at least 30% impaired binding to the E1198A mutant, and IgG from 10 or 11 patients showed impaired binding to CFH_19–20_ mutants T1184R, K1186A, and K1188A compared with WT CFH_19–20_ (Fig. 2*B*). IgG from one to three patients showed impaired binding to mutants D1119G, Q1139A, R1182A, W1183L, and R1210A, whereas IgG from all patients showed similar binding to WT CFH_19–20_ and mutants W1157L, R1203A, R1206A, and R1215Q (Fig. 2*B*).

We also found that binding of mAb C18, which has previously been shown to have an epitope overlapping with that of several CFH-AAs (11, 23), to the K1186A mutant was diminished (Fig. 2*A*). This antibody efficiently inhibited binding of CFH-AA to CFH_19–20_. This inhibition was also observed in those cases (patients 10, 11, 12, and 14) where the autoantibodies showed only moderately impaired binding to the five CFH mutants within the common autoantibody binding site (data not shown). This indicates that the main binding site of the autoantibodies from those patients is likely to be overlapping.

##### The Autoantibody-binding Site Overlaps with the Heparin- and Common Microbe-binding Sites

Our previously solved crystal structure of CFH_19–20_ (7) was used to visualize the location of the five residues of CFH_19–20_ (Thr-1184, Lys-1186, Lys-1188, Leu-1189, and Glu-1198) that were found to be involved in binding of CFH-AAs from at least 10 of 17 patients. These residues form a tightly packed cluster (diameter of ∼11 Å) on one side of CFH domain 20 termed the CFH-AA site (Fig. 2*C*). Compared with the previously described functional sites on CFH_19–20_, the CFH-AA site is clearly distinct from the two sites for C3b or C3d on domains 19 and 20 (6, 21) but adjacent to and partially overlapping with the site involved in binding of CFH to heparin and endothelial cells (Fig. 2*D*) (5, 40), as well as with the recently described common microbe-binding site on CFH domain 20 (Fig. 2*E*) (29, 30).

##### Binding of CFH-AAs to the CFHR1 C Terminus

**FIGURE 3.**
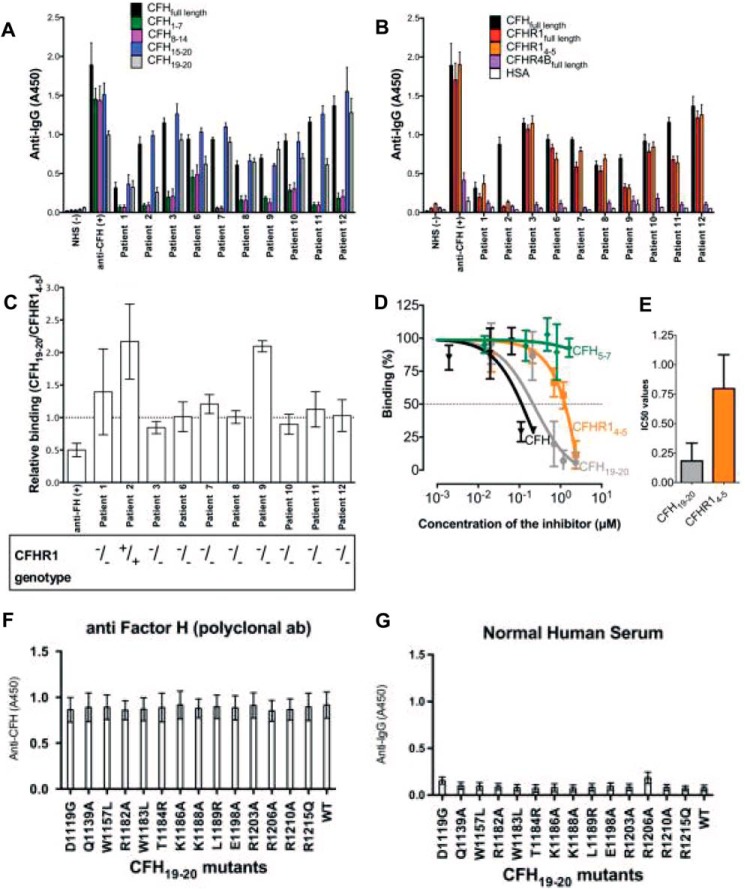
**Binding of autoantibodies from autoimmune aHUS patients to CFH and CFHR1.** *A*, binding of autoantibodies to CFH (full-length) and its fragments CFH_1–7_, CFH_8–14_, CFH_15–20_, and CFH_19–20_. *B*, binding of IgG autoantibodies to CFH, CFHR1 (full-length), CFHR1_4–5_, and CFHR4B (full-length). Human serum albumin (*HSA*) and/or normal human serum (*NHS*) was used as a negative control, and goat anti-CFH polyclonal antibody was used as a positive control. *C*, bar diagram elucidating the relative binding ratio of the patient autoantibodies to CFH_19–20_ and CFHR1_4–5_. *Error bars* indicate S.E. The CFHR1 deficiency of each patient is shown below. *D*, the binding of purified IgG from patient 11 to CFH_19–20_ was tested in the presence of increasing concentrations of CFH_19–20_ or CFHR1_4–5_. CFH_5–7_ was used as a negative control. *E*, bar diagram of the concentration of CFH_19–20_ or CFHR1_4–5_ needed for 50% inhibition (*IC50*) obtained from three independent experiments performed in triplicates. *F*, binding of anti-CFH polyclonal antibody to the CFH_19–20_ mutants. *G*, no binding was noticed between CFH_19–20_ mutants and IgG from normal human serum. *Error bars* indicate S.E.

On the basis of the previously solved structure of CFH_19–20_, the two residues that are different in CFH_19–20_ and CFHR1_4–5_ (Ser-1191 and Val-1197 of CFH) are buried beneath the identified CFH-AA site. Therefore, we studied whether the difference of the two amino acids in the C-terminal domains of CFH and CFHR1 has an influence on the binding of autoantibodies from patient sera. The level of binding of antibodies from patient sera to both CFH_19–20_ and CFHR1_4–5_ varied from patient to patient, and this seemed to correlate to the antibody titer in the sera (Table 1). IgG from 8 of the 10 CFH-AA patient samples of which we had enough available for analysis bound similarly to CFHR1_4–5_, CFH, and CFH_19–20_, whereas binding of autoantibodies from two of the samples (patients 2 and 9) to CFHR1_4–5_ was diminished (Fig. 3, *A–C*). Next, a purified IgG fraction from patient 11 was used to compare the affinities of CFH-AAs to CFH_19–20_ and CFHR1_4–5_ by inhibition assay. Binding of IgG to CFH was somewhat stronger in this assay (Fig. 3, *D* and *E*), although a clear difference in binding to CFH_19–20_ and CFHR1_4–5_ could not be detected in the ELISA assay (Fig. 3, *B* and *C*). Taken together, the results on the difference in binding of CFH-AAs to CFH_19–20_ and CFHR1_4–5_ indicated that the conformation of the CFH-AA site needs to be slightly different in CFH domain 20 and CFHR domain 5.

**TABLE 1:** **CFH-AA-positive patient sample data** AU, arbitrary units.

Patient	*CFHR1* genotype	Sampling times	Ig subtype	Light chain type	CFH-AA titer
					*AU*
Patient 1	−/−	Convalescence	IgG3	λ	456
Patient 2	+/+	Convalescence	IgG1, IgG3	κ	868
Patient 3	−/−	Acute phase	IgG1, IgG3	κ	1325
Patient 4	−/−	Convalescence	IgG3	λ	1067
Patient 5	−/−	Acute phase	IgG1, IgG3	λ	1043
Patient 6	−/−	Acute phase	IgG3	λ	1136
Patient 7	−/−	Convalescence	IgG3	λ	1132
Patient 8	−/−	Convalescence	IgG3	λ	883
Patient 9	−/−	Convalescence	IgG1	κ	1063
Patient 10	−/−	Convalescence	IgG3	λ	769
Patient 11	−/−	Acute phase	IgG3	λ	3548
Patient 12	−/−	Convalescence	IgG1, IgG3	λ	1239
Patient 13	−/−	Acute phase	IgG1, IgG3	κ, λ	1260
Patient 14	−/−	Convalescence	IgG3	κ	737
Patient 15	−/−	Convalescence	IgG3	λ	444
Patient 16	−/−	Convalescence	IgG1, IgG3	κ, λ	671
Patient 17	−/−	Convalescence	IgG3	λ	852

##### Structures of the C Termini of CFHR1 and CFH Are Nearly Identical

**FIGURE 4.**
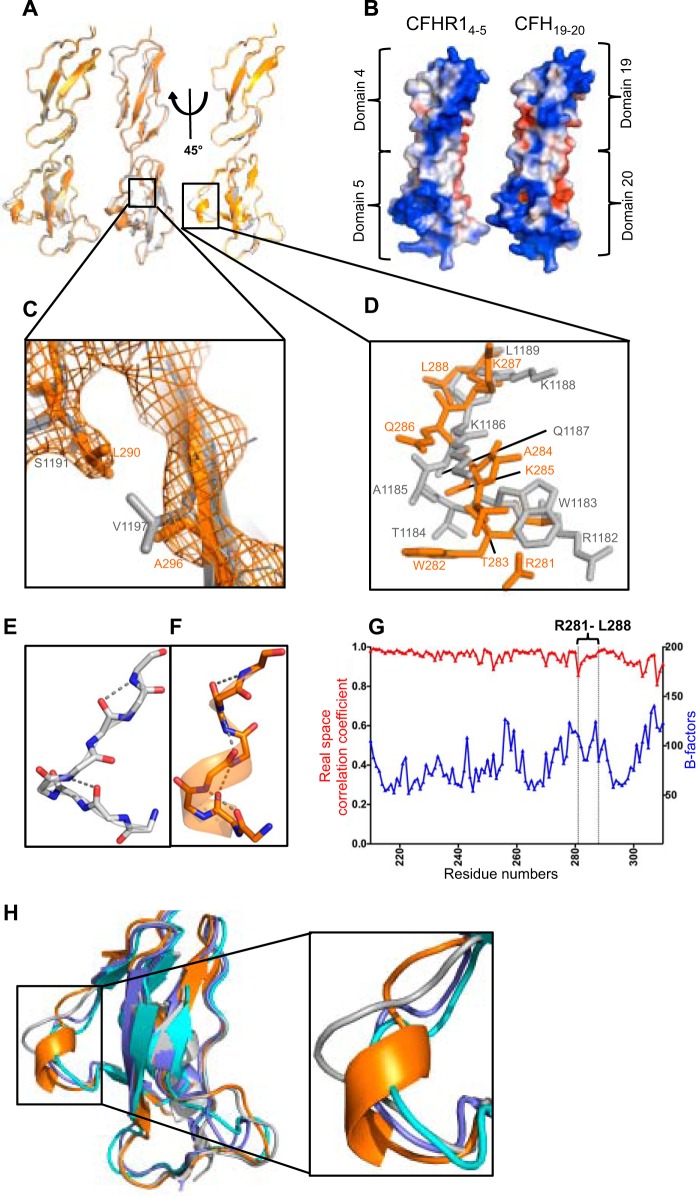
**Crystal structure of CFHR1_4–5_ and comparison with the previously solved structure of CFH_19–20_.** *A*, structural superposition of the two molecules of CFHR1_4–5_ (*orange* and *yellow*) found in the asymmetric unit along with CFH_19–20_ (*gray*) shown in a *cartoon representation. B*, comparison of the surface charge potentials of CFHR1_4–5_ and CFH_19–20_. Potentials on the solvent-accessible surfaces were calculated and displayed at the ±2 *kT*/e level on both structures after modeling all of the missing side chains of the previously published structure of CFH_19–20_ (Protein Data Bank code 2G7I) (7). *C*, close-up view of the two residues that are different in the amino acid sequences of these two protein constructs with the 2m*F_o_* − D*F_c_* electron density map of CFHR1_4–5_ shown. *D*, close-up view of the region in which the tertiary structures of CFHR1_4–5_ and CFH_19–20_ are dissimilar (Arg-1182–Leu-1189 of CFH_19–20_ and the corresponding Arg-281–Leu-288 of CFHR1_4–5_) with backbone and side chain atoms shown as a stick model. This region corresponds to the CFH-AA-binding site shown in Fig. 2*C*. The hydrogen bonds found in the autoantigenic loop of CFH (*E*) and its homologous region in CFHR1 (*F*) that stabilize the structure. *G*, comparison of the real space correlation constants and *B*-factors of the residues of CFHR1_4–5_. The *B*-factors are indicated on the *right y axis*, and the real space correlation coefficients are indicated on the *left y axis*. The CFH-AA site (Arg-281–Leu-288) is indicated. *H*, *cartoon representation* of the structural superposition of CFHR1_4–5_ (*orange*; Protein Data Bank code 4MUC) with CFH_19–20_ (*gray*; code 2G7I (7)), CFH_19–20_ in complex with a sialic acid glycan and C3d (*slate*; code 4ONT (22)), and CFH_19–20_ in complex with OspE (*turquoise*; code 4J38 (29)).

To detect possible differences in the CFH-AA site on CFH_19–20_ and the corresponding site on CFHR1, we used x-ray crystallography to solve the structure of CFHR1_4–5_. The structure was obtained as a homodimer at 2.9 Å resolution from a different space group (*P*622) than the previously published CFH_19–20_ structures (*I*4_1_22) (Table 2) (7, 28), but the structures aligned very well with each other (Fig. 4*A*). Superimposing the CFHR1_4–5_ structure with the previously published structures of CFH_19–20_ solved as homotetramers (7), in complex with C3d (6), or in complex with the borrelial OspE protein (29) gave a root mean square deviation (r.m.s.d.) of 0.5–1.1 Å for the 113 aligned Cα atoms, indicating that the structures are nearly identical. Also, the charge potentials on the solvent-accessible surface displayed at the ±2 *kT*/e level on CFHR1_4–5_ and CFH_19–20_ were similar all around the molecules (Fig. 4*B*).

**TABLE 2:** **Data collection and refinement statistics for CFHR1_4–5_** Statistics for the highest resolution shell are shown in parentheses.

Resolution range (Å)	48.53–2.897 (3.001–2.897)
Space group	*P*622
Unit cell dimensions	
*a*, *b*, *c* (Å)	143.406, 143.406, 77.784
α, β, γ	90°, 90°, 120°
Unique reflections	10,935 (1048)
Completeness (%)	99.78 (98.59)
Mean *I*/σ*I*	15.30 (2.46)
Wilson *B*-factor	72.41
*R*_work_	0.2070 (0.3078)
*R*_free_	0.2683 (0.3642)
No. of atoms	1939
Macromolecules	1904
Ligands	35
Water	3
Protein residues	248 (both molecules in the asymmetric unit)
r.m.s.d.	
Bond lengths (Å)	0.009
Bond angles	1.16°
Ramachandran favored (%)	95
Ramachandran outliers (%)	0
Average *B*-factor	96.4
Macromolecules	95.5
Solvent	80.4

##### Differences Noticed in the Structure of the CFH-AA Site of CFH_19–20_ and CFHR1_4–5_

The structures of CFH_19–20_ and CFHR1_4–5_ are seemingly identical, but the detailed tertiary structure of the buried region containing mutations S1191L and V1197A is naturally somewhat different (Fig. 4*C*). In addition, as expected on the basis of the differences observed in binding of certain autoantibodies and the previously reported functional differences of CFH/CFHR1 hybrid proteins, also the conformation of the CFH-AA site on CFHR1_4–5_ is slightly different from that on CFH_19–20_ (r.m.s.d. of the backbone atoms of the loop region = 3 Å). Both the backbone and loops forming the CFH-AA site (Arg-281–Leu-288 of CFHR1 and Arg-1182–Leu-1189 of CFH, respectively) have a different orientation (Fig. 4*D*). The main difference in the backbone is the formation of a short α-helix in the loop of CFHR1_4–5_, whereas no prominent helix is seen in this region in any of the solved structures of CFH_19–20_. The orientation of some of the side chains is also clearly different, as Arg-281–Leu-288 of CFHR1_4–5_ are distinctively apart from the location of the side chains of the corresponding residues of CFH_19–20_ (Arg-1182–Leu-1189) (Fig. 4*D*). Also, the hydrogen bonds stabilizing the loop in CFH and CFHR1 are clearly different (Fig. 4, *E* and F). The real space correlation coefficients and the *B*-factors of the loop residues show normal behavior (Fig. 4*G*), and there are no crystal contacts within this region in either the CFHR1_4–5_ or CFH_19–20_ structure. Interestingly, only one of the two CFHR1_4–5_ molecules in the same crystalline space shows a conformation that is dissimilar to the CFH-AA site on CFH domain 20, whereas the other CFHR1_4–5_ molecule has a site that is similar to CFH.

Because the data indicated that the Arg-281–Leu-288 loop of CFHR1 is flexible (or has two conformations), we next analyzed whether flexibility is observed in the CFH-AA site of the previously published structures of CFH_19–20_. The conformation of the Arg-1182–Leu-1189 loop is somewhat different in free CFH_19–20_ compared with the mutually similar conformation of CFH_19–20_ in complex with either the microbial protein OspE (r.m.s.d. of the backbone of the loop residues = 2.7 Å) or the natural ligands C3d (r.m.s.d. of the backbone of the loop residues = 3.0 Å) or sialic acid glycan (r.m.s.d. of the backbone of the loop residues = 2.9 Å) (Fig. 4*H*). This conformation is also different from that of CFHR1 (r.m.s.d. of the backbone of the loop residues = 3.0 Å). This indicates the structural flexibility of the CFH-AA site upon binding of a ligand to the same domain (Fig. 4*H*).

**FIGURE 5.**
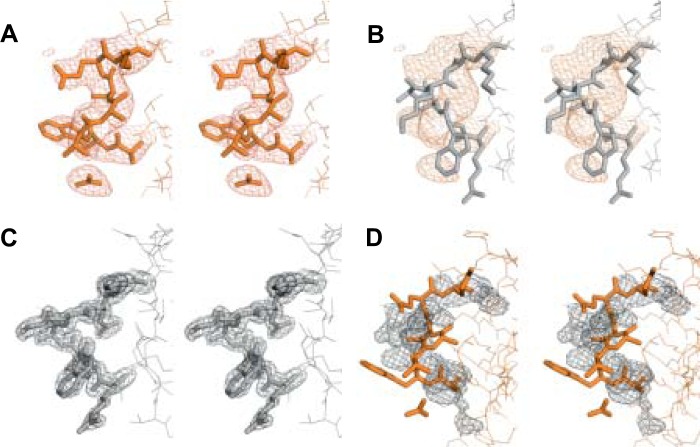
**Comparison of the models and 2m*F_o_* − D*F_c_* electron density maps of the CFH-AA-binding site of CFH_19–20_ and the corresponding site of CFHR1_4–5_.** The CFHR1_4–5_ loop region (Arg-281–Leu-288; *A*) and the CFH_19–20_ loop region (Arg-1182–Leu-1189; *B*) are shown with the CFHR1_4–5_ 2m*F_o_* − D*F_c_* electron density map. The CFH_19–20_ loop region (Arg-1182–Leu-1189; *C*) and the CFHR1_4–5_ loop region (Arg-281–Leu-288; *D*) are shown with the CFH_19–20_ 2m*F_o_* − D*F_c_* electron density map. A stick model and electron density map of CFHR1_4–5_ are displayed in *orange*, and those of CFH_19–20_ in *gray*.

To exclude potential misinterpretation of the x-ray diffraction data of the CFH-AA site, we next compared the electron density maps (2m*F_o_* − D*F_c_*) of Arg-1182–Leu-1189 of CFH_19–20_ (7) and Arg-281–Leu-288 of CFHR1_4–5_ (Fig. 5). Clearly, the model of CFHR1_4–5_ (but not CFH_19–20_) fits very well with the electron density map of CFHR1_4–5_ in this region (Fig. 5, *A* and *B*), whereas the corresponding region in the model of CFH_19–20_ (but not CFHR1_4–5_) fits well with the electron density map of CFH_19–20_ (Fig. 5, *C* and *D*).

## DISCUSSION

Autoimmune aHUS is an unusual autoimmune disease because it is associated with a deficiency of a protein (CFHR1) homologous to the autoantigen (CFH). Therefore, it offers an exceptional opportunity to study phenomena leading to antibody-associated autoimmunity. This study shows that the amino acid residues contributing to the binding sites of CFH-AAs from 17 patients with autoimmune aHUS form a cluster on domain 20 of CFH adjacent to the common microbe-binding site. The differential binding of CFH-AAs from two aHUS patients to CFH_19–20_ and CFHR1_4–5_, as also suggested previously (13, 26), and the small but clear differences in the x-ray crystal structures of the loop forming the autoantigenic epitope on CFH and CFHR1 indicate that the C-terminal domain of CFH and CFHR1 can have slightly different conformations. In addition, the conformation of the autoantigenic loop on CFH seems flexible because we noticed that the loop conformation is slightly different after binding of ligands to domain 20 of CFH in the previously published structures (22, 29). The reason for the association of CFHR1 deficiency with aHUS is unexplained, but our results enabled us to evaluate previous results and explanation models and to generate a new hypothesis of an induced autoantigenic neoepitope. This explains the association between CFHR1 deficiency and autoantibody formation against the common CFH-AA epitope on domain 20.

aHUS-associated mutations in the C-terminal domains of CFH have been shown to cause reduced binding of CFH to C3b or host cell-surface structures such as glycosaminoglycans/heparin (5–7, 41–43). aHUS-associated autoantibodies to CFH_19–20_ cause uncontrolled complement attack against host cells, and autoantibodies from some patients have been shown to impair CFH binding to C3b or to host cells (11). The location of the CFH-AA-binding site we identified on domain 20 indicates that the autoantibodies are likely to block binding of CFH at least to glycosaminoglycans/heparin due to the proximity of the CFH-AA site to the heparin-binding site. In addition, the location of the hemolysis-inducing aHUS mutation W1183L (26, 44) next to the CFH-AA site (Figs. 2*C* and 4*D*) indicates the importance of the site in protecting host cells from complement. Although both the C3b-binding sites on CFH_19–20_, one on domain 19 and the other on domain 20 (6, 21), are relatively distant from the CFH-AA-binding site, the CFH-AAs might interfere with C3b binding due to their large size, as has been reported with mAb C18, which we now (Fig. 2*A*) and previously found (11) to bind to the same region as the CFH-AAs. Diminished binding of CFH to either the cell-surface structures or C3b can lead to compromised protection of the plasma-exposed host cells, which has been reported widely in aHUS patients (1).

Close comparison of the conformation of the autoantigenic loop of CFH in the published crystal and NMR structures of CFH_19–20_ (6, 7, 21, 22, 29, 40, 45) shows minor differences in the Arg-1182–Leu-1189 region of CFH_19–20_ in complex with OspE (29), C3d (6), or a sialic acid glycan (22). The r.m.s.d. of the backbone atoms of the loop region is 2.7–3.0 Å compared with CFH_19–20_ alone (Fig. 4*H*). Binding of CFH_19–20_ to heparin tetrasaccharide has also been shown to cause chemical shift perturbations in NMR at the CFH-AA site (22, 40). Thus, it is likely that the conformation of the region is somewhat flexible. It seems possible that the corresponding site on CFHR1 is also structurally flexible because of the two monomers in the unit cell of the CFHR1_4–5_ crystal lattice, conformation of the CFH-AA site of one monomer was similar to CFH, and the other one was different (Fig. 4*A*). The key difference between CFH and CFHR1 could therefore be that CFHR1 takes the alternative conformation spontaneously, whereas on the basis of various crystal and NMR structures, CFH_19–20_ takes a slightly altered conformation only upon binding of a ligand. However, we do not suggest that binding of self-molecules (such as heparin, C3b, or sialic acids) leads to autoimmunity, but the flexibility of the autoantigenic loop upon binding of these ligands to CFH domain 20 is obvious.

The observed difference between the CFHR1_4–5_ and the CFH_19–20_ structures is unlikely to be a crystallographic artifact due to the four following reasons. First, only one of the monomers in the unit cell containing two CFHR1_4–5_ molecules shows a structure that is considerably different from CFH domain 20 (see Protein Data Bank code 4MUC), indicating structural flexibility in that loop of CFHR1 domain 5. Second, there are no direct contacts between the residues of the CFH-AA site (Arg-281–Leu-288) and the molecule in the neighboring crystal cell. Third, CFH-AAs from two of the studied 10 patients bound differently to CFH_19–20_ and CFHR_4–5_ (Fig. 3*C*), indicating that there is a difference within the CFH-AA-binding site of these molecules. Fourth, CFHR1_4–5_ and CFH_19–20_ have been reported to have functional differences (45), which is obvious because the fusion proteins CFH_1–18_/CFHR1_4–5_ and CFHR1_1–3_/CFH_19–20_ are associated with aHUS and have different functions compared with normal full-length CFH and CFHR1 (4, 18–20). Because domain 19 of CFH and domain 4 of CFHR1 are identical, it has been deduced that the difference leading to the clinical disease is within the terminal domain of the fusion proteins, leading to the inability to control complement on self-surfaces (19). Our results suggest that the reason for the functional difference between the most C-terminal domains of CFH and CFHR1 is their varied ability to bind to heparin or glycosaminoglycans on self-cells because the loop that has a different conformation in CFH and CFHR1 contains several of the heparin-binding residues (Fig. 2, *C* and *D*; and Fig. 4*D*) (5, 40).

Nearly all patients with CFH-AAs lack CFHR1 (12, 13); thus, it is likely that the absence of CFHR1 imparts the risk of CFH-AA generation. The risk for anti-CFH autoimmunity in the absence of CFHR1 is very high, as the odds ratio is 442 (16). In this study, we provided data for a structure-based molecular explanation of the phenomenon. The explanation is based on four observations from our study. First, the binding sites of CFH-AAs from the 17 patients analyzed clearly formed a cluster, the CFH-AA site. Second, the CFH-AA site in CFH domain 20 is adjacent to the two buried residues (Ser-1191 and Val-1197) that are different between CFH_19–20_ and CFHR1_4–5_. Third, although the crystal structures of CFHR1_4–5_ and CFH_19–20_ are similar due to the 98.5% sequence identity between them, there is a small structural difference exactly at the CFH-AA site on the Arg-1182–Leu-1189 loop of CFH (Fig. 4*D*). Fourth, a small conformational change has been detected within the autoantigenic loop upon binding of CFH_19–20_ to a microbial protein (29), a sialic acid glycan (22), heparin, or C3d.

**FIGURE 6.**
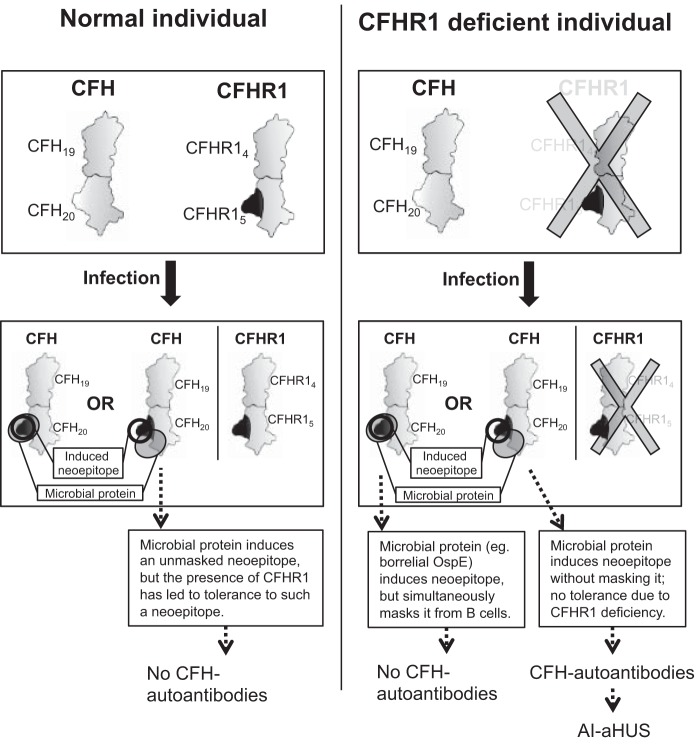
**Schematic illustration of a model to explain the occurrence of CFH-AAs in aHUS, with special attention to the strong association of autoimmune aHUS with homozygous deficiency of CFHR1.** *Upper panels*, the phenotypes of normal and CFHR1-deficient individuals are shown schematically to indicate the structural difference observed between the CFH-AA site and the corresponding site on CFHR1 (*dark gray* protrusion). In the novel “induced neoepitope model,” binding of a microbial protein to CFH domain 20 (adjacent to the autoantigenic Arg-1182–Leu-1189 loop, thus not masking it from B-cells) induces a conformational change in the Arg-1182–Leu-1189 loop of domain 20, thereby making its conformation similar to that of CFHR1 domain 5.

The two usual models to explain autoantibodies in general, an analogous epitope (molecular mimicry) (46) and a co-epitope (formed by adjacent molecules) (47), are unable to explain the association between CFHR1 deficiency and CFH-AA binding to the CFH-AA site on domain 20. Thus, on the basis of the new data, we propose a novel explanation for the association of CFHR1 deficiency with the autoimmune disease. In this model, called the “induced neoepitope model,” the normal structure of the Arg-1182–Leu-1189 loop of CFH can be turned into an autoantigenic conformation upon induction by at least one microbial ligand binding to that region of CFH domain 20 (Fig. 6). It has been reported that several kinds of infections can precede autoimmune aHUS, and this is concordant with our model because several microbial molecules are known to bind close to the autoantigenic epitope of CFH (29, 30), and we observed from previously published data that CFH_19–20_ in complex with OspE (29) has a slightly altered conformation in this region.

It is possible that binding of certain microbial ligands to the autoantigenic loop or close to it leads to masking of the autoantigenic epitope. We reported previously that several microbes bind next to the autoantigenic loop (30). Therefore, all of these may induce the autoantigenic neoepitope, but some of them could simultaneously mask the loop. This is the case at least with borrelial OspE, which binds directly to the autoantigenic loop (29). It is noteworthy that borreliosis has never been reported preceding autoimmune aHUS. Therefore, we do not suggest that all microbial molecules that bind to CFH domain 20 could lead to autoimmunity in CFHR1-deficient individuals. However, it is possible that some of the microbial molecules that bind to the domain do not mask the autoantigenic loop, thereby leading to the risk of autoimmunity in the absence of CFHR1 (Fig. 6).

Our model provides an explanation as to why immunization against the CFH-AA site could occur only in CFHR1-deficient individuals because, in normal individuals, the presence of CFHR1 with an epitope similar to the hypothetical induced autoantigenic conformation of CFH would have guaranteed tolerance to that conformation of CFH. The model proposed here also explains the other four key biological phenomena described in autoimmune aHUS. First, the association with infections (14, 48). Second, the clustering of the autoantibody epitopes on CFH domain 20 (Fig. 2). Third, the high prevalence of IgG or IgA class autoantibodies (9, 26) because the foreign peptide needed for class switch of B-cells by T-cell help could be provided by the microbial protein bound to CFH_19–20_. And finally, the model explains the polyclonality of the autoimmune response (15) because different epitopes on the autoantigenic loop could be recognized by various B-cell receptors.

In this study, we have shown that CFH-AAs bind to a common site on the Arg-1182–Leu-1189 loop of CFH next to the two buried residues that are different in CFH_19–20_ and CFHR1_4–5_. The crystal structure of CFHR1_4–5_ presented here shows that the conformation of the autoantigenic loop is different in CFH and CFHR1. Taken together, these data provided the basis for the suggested novel model (Fig. 6) to explain how CFHR1 deficiency is linked to CFH-AA formation.

## Footnotes

3 The abbreviations used are:

aHUS: atypical hemolytic uremic syndrome
CFH: complement factor H
CFH-AA: anti-CFH autoantibody
r.m.s.d.: root mean square deviation.

## Note Added in Proof

Tables 1 and 2 were numbered incorrectly in the version of this article that was published on February 6, 2015 as a Paper in Press. The error has been corrected.
